# Rhythm control in persistent atrial fibrillation improves endothelial function without uniform anti-inflammatory effects: A 9-month prospective cohort study

**DOI:** 10.1016/j.ijcha.2026.101879

**Published:** 2026-01-23

**Authors:** Maximilian Seidel, David Bogdahn, Felix S. Seibert, Moritz Anft, Sarah Skrzypczyk, Ulrik Stervbo, Eva Kohut, Kamil Rosiewicz, Benjamin Sasko, Christian Ukena, Nina Babel, Timm H. Westhoff

**Affiliations:** Medizinische Klinik I, Universitätsklinik Marien Hospital Herne, Ruhr-Universität Bochum, Hölkeskampring 40, 44625 Herne, Germany

**Keywords:** Atrial fibrillation, Endothelial function, Inflammation, Cytokines

## Abstract

**Background:**

Atrial fibrillation (AF) is associated with systemic inflammation, endothelial dysfunction, and adverse cardiovascular outcomes. While there is robust evidence, that inflammation contributes to AF pathogenesis, the existence of a reverse relation – whether AF contributes to inflammation − remains elusive. This study therefore evaluates the impact of rhythm control on systemic inflammation and endothelial function.

**Methods:**

In this prospective observational study, 124 patients with persistent AF undergoing successful rhythm control therapy (electrical cardioversion or catheter ablation) were followed for nine months. Various Cytokines, high-sensitivity C-reactive protein (hsCRP), flow-mediated dilation (FMD) and additional inflammatory biomarkers were measured at baseline, 1 week, 1 month, 3 months, and 9 months. FMD was assessed by high-resolution brachial artery ultrasound. Patients with AF recurrence throughout the follow-up period were excluded from primary analysis.

**Results:**

In patients without AF recurrence, FMD improved significantly from 6.3 % (4.6–8.3) to 7.6 % (5.1–8.8) (p < 0.001). HsCRP, IL-8 and fibrinogen declined modestly (p = 0.002, p < 0.001 and p < 0.001, respectively), whereas IL-2, IP-10, IL-12p70, MCP-1 and TNF-α increased significantly over time (all p < 0.001). Elevated pre-treatment hsCRP was weakly associated with AF recurrence (r = 0.20, p = 0.023; AUC = 0.61). IL-6 showed temporal variation but no sustained change from baseline.

**Conclusion:**

Rhythm control therapy in persistent AF is associated with an improvement of endothelial function but not with a homogeneous improvement of systemic inflammatory serological profiles. Thus, the improvement in FMD appears to be mediated primarily by hemodynamic restoration rather than anti-inflammatory effects.

## Highlights

• Rhythm control in atrial fibrillation improves endothelial function.
• Endothelial recovery occurs without consistent anti-inflammatory effects.
• Inflammatory cytokines show heterogeneous patterns after rhythm control therapy.
• Improvement in vascular health appears driven by hemodynamic normalization.

## 1 Introduction

Atrial fibrillation (AF) is the most prevalent sustained cardiac arrhythmia worldwide. AF remains a serious public health burden. Its incidence and prevalence continue to rise, driven largely by an aging population, leading to serious complications such as heart failure, cognitive impairment, thromboembolism and death [1]. In most cases AF does not result from a single identifiable cause, but rather arises from a multifactorial pathogenesis, with various contributing factors leading to its onset or exacerbation, such as hypertension, heart failure, valvular heart disease and systemic inflammation [2,3]. In recent years, there has been growing interests investigating the role of systemic inflammation as a contributing factor to AF. This interest arose from the strong pathophysiological link between AF and systemic inflammation. Proposed pathomechanisms include cytokine-mediated atrial and electrical remodelling, oxidative stress leading to damage of atrial tissue and alterations in autonomic nerve function toward AF-promoting states [[4], [5], [6], [7]]. While there is an increasing number of trials confirming inflammation as a contributing factor to AF, there is only little data on the reverse relationship, specifically how rhythm control therapy influences the level of systemic inflammation. Patients with AF are known to exhibit elevated systemic inflammation compared to individuals without AF [3].

Fittingly, systemic inflammation is associated with an impairment of endothelial function, underlining its cardiovascular significance [8]. Endothelial function, particularly as assessed by flow-mediated dilatation (FMD), provides insight into nitric oxide (NO)-dependent vasodilation and serves as both a key indicator of vascular health and a predictor of cardiovascular risk [9,10]. Endothelial dysfunction is considered one of the earliest signs of atherosclerosis. In the context of atrial fibrillation (AF), the relationship with endothelial dysfunction appears to be bidirectional: while evidence supports the hypothesis that endothelial dysfunction may precede AF development, further findings suggest it may also arise as a consequence of AF [11,12]. Chronic systemic inflammation is a key pathophysiological driver in the pathogenesis and worsening of many degenerative, metabolic and cardiovascular diseases. It is therefore crucial to limit the level of chronic inflammation, intending cardiovascular risk reduction. Data on systemic inflammation post rhythm control are sparse and divergent [13,14]. While successful ECV has been associated with lower levels of high-sensitive CRP (hsCRP), multiple studies have reported an increase in systemic inflammation following catheter ablation procedures [[15], [16], [17]].

This study aims to determine whether rhythm control therapy reduces systemic inflammation, thereby supporting a bidirectional relationship between inflammation and atrial fibrillation. Furthermore, it evaluates the impact of rhythm control on endothelial function.

## 2 Methods

### 2.1 Study population and study design

We conducted a prospective observational study investigating systemic inflammation and endothelial function following rhythm control therapy in patients with atrial fibrillation.

Inclusion criteria were persistent atrial fibrillation with indication for rhythm therapy either by ECV or catheter ablation. Exclusion criteria were hemodynamically instability, paroxysmal or permanent AF, C-reactive protein (CRP) > 5 mg/dl and age < 18 years. We identified n = 124 patients that received successful rhythm control therapy between November 2021 and February 2024. The study protocol included analysis of cytokines and further serological inflammatory markers, measurements of FMD and documentation of relevant epidemiological data during the follow-up period in a German University Hospital. The observation period for the treatment group spanned 9 months. Measurements were conducted the day before rhythm control therapy, as well as at 1 week, 1 month, 3 months, and 9 months post-treatment. AF recurrence during follow-up was assessed by scheduled 12-lead electrocardiograms at each study visit and additional ECG recordings obtained during routine clinical follow-up or symptom-driven evaluations. Holter monitoring was performed when clinically indicated. AF recurrence was defined as any documented episode of atrial fibrillation after the blanking period. Patients with AF recurrence were excluded from FMD and cytokine analysis to specifically evaluate the effects of sustained sinus rhythm on endothelial function and inflammatory markers. Given the mechanistic focus of the study, an intention-to-treat approach was not pursued. Informed consent was obtained from each patient. The study protocol conforms to the ethical guidelines of the 1975 Declaration of Helsinki as reflected in a priori approval by the institution's human research committee (Reg. No**.:** 15–5279 Ethics committee of the Ruhr-University Bochum).

### 2.2 Flow-mediated dilation measurement

As published previously, FMD measurement is performed on the dominant arm using ultra sound (Aloka/Hitachi, Prosound alpha 6) with a high resolution (≥7.5 MHz) linear-array transducer to display the brachial artery in the cubital area. The examinations were performed under standard conditions (room temperature 22 °C, quiet environment). Blood pressure was measured by an oscillometric method before initiation of FMD measurement. Participants were positioned in a supine position for 15 min to avoid a potential effect of stress and they were instructed not to speak during the examination. Assessment of endothelium-dependent vasodilation was done at the level of right brachial artery 5–10 cm above the antecubital fossa, according to the recommendations for FMD assessment [18,19]. The software’s tracking system followed the motion of the vessel walls caused by pulsations and automatically measured the change in vessel diameter with a precision up to 0.01 mm in real time which has been shown to reduce inter-observer variability compared with manual diameter assessment. The waveform of diameter changes over the cardiac cycle was displayed in real time using the FMD-mode of the eTRACKING system. The internal diameter of the right brachial artery was continuously monitored using the following protocol: 1 min recording of baseline diameter at rest, 5 min recording during a forearm ischemia induced by inflation of a pneumatic forearm cuff 50 mmHg above systolic blood pressure, and 3 min of post-deflation diameter recording. All examinations were performed by the same specialist trained in 2D and Doppler ultrasonography. To minimize operator-dependent error, a mechanical probe holder was used with the fixed angle approximately 60° between the probe and the vessel orientation in all the examinations. To further minimize measurement variability, all FMD assessments were performed by a single experienced operator. Although formal inter-observer reliability testing was not applicable due to the single-operator design, repeated measurements followed standardized international guidelines. There is currently no universally accepted cut-off value for FMD. In a *meta*-analysis by Heiss et al. (2023), an FMD ≥ 6.5 % was identified as a threshold above which coronary artery disease could be excluded with high sensitivity (95 %) [20]. Maruhashi et al. proposed a cut-off value of FMD ≥ 7.1 % to indicate normal endothelial function in a large Japanese cohort [21]. For the present study, we referred to the European cohort analysis by Heiss et al. and defined FMD as normal when ≥ 6.5 %.

### 2.3 Cytokine analysis

Serum samples were collected into a S-Monovette Serum collection tubes (Sarstedt, Germany), stored at −20 °C and then assessed for the levels of cytokines IL-4, IL-2, IP-10 (CXCL10), IL-1β, TNF-α, MCP-1 (CCL2), IL-17A, IL-6, IL-10, IFN-γ, IL-12p70, L-8 (CXCL8), and TGF-β1 (free active) using the LEGENDplex Human Essential Immune Response Panel (BioLegend, San Diego, USA) and acquired on a CytoFLEX flow cytometer (Beckman Coulter, Germany). Cytokine concentrations were quantified using a bead-based flow cytometric assay according to the manufacturer’s instructions.

### 2.4 Statistical analysis

Data were tested for normality using the Kolmogorov–Smirnov test. Normally distributed variables are presented as mean ± standard deviation (SD), while non-normally distributed variables and categorical data are reported as median and interquartile range (IQR). Within-group comparisons from baseline to follow-up were conducted using a paired, two-tailed *t*-test for normally distributed data. Comparisons between two independent groups were performed using an unpaired *t*-test. For comparisons across more than two follow-up time points, one-way analysis of variance (ANOVA) was used for normally distributed data, and the Friedman test was applied for non-normally distributed data. Categorial parameters were analyzed using X2-Test. Correlation analyses were performed using Pearson’s correlation for parametric data and Spearman’s rank correlation for nonparametric data. To explore independent associations with AF recurrence, a multivariable logistic regression model was fitted including baseline hsCRP, IL-8, and TGF-β as candidate predictors based on their baseline group differences. Linear mixed-effects models were used to assess longitudinal changes in FMD across follow-up time points, adjusted for body mass index, statin therapy, and antihypertensive medication. Given the exploratory nature of this study, corrections for multiple testing were not applied. All statistical analyses were done using SPSS Statistics 29 (SPSS Inc, Chicago, Illinois, USA), GraphPad Prism 10 (GraphPad Software Inc., Boston, Massachusetts, USA) and MedCalc 23.2 (MedCalc Software Ltd., Osend, Belgium).

## 3 Results

The patients’ characteristics are presented in Table 1. Briefly, the average age at the time of inclusion was 70 ± 11 years across all participants. Average body mass index (BMI) was measured at 30.0 ± 5.0  kg/m2, Females comprised 44.4 % of the total study population. Hypertension emerged as the most common comorbidity, affecting 87.9 % of. This was followed by hypercholesterinemia, which was present in 45.2 %. Coronary heart disease was reported in 39.5 %. In terms of antihypertensive therapy, beta blockers were the most frequently prescribed medication, taken by 83.1 % of all participants. ACE inhibitors and AT1 antagonists were prescribed in 31.5 % and 41.1 % of cases, respectively. The mean number of antihypertensive agents used was 2.6 ± 1.4 overall. Statin therapy was prevalent in 68.5 % of the patients. Recurrence of atrial fibrillation occurred significantly more frequently following electrical cardioversion compared to catheter ablation (15 of 68 (22.1 %) vs. 5 of 56 (8.9 %), p = 0.048). 43 patients (34.7 %) experienced a recurrence of atrial fibrillation during the observational period and were excluded from the study in accordance with the predefined exclusion criteria. Mean time of observation were 8.1 ± 2.5 months.

**Table 1:** Epidemiological and clinical data of the study population. BMI: body mass index, ECV: electrical cardioversion, RFA: radiofrequency catheter ablation, ACE: *A*ngiotensin converting enzyme, AT1: angiotensin receptor 1, MRA: mineralocorticoid receptor antagonists, PCSK-9-I: proprotein convertase subtilisin/kexin type 9.

		Patients (n = 124)
Age [years]		70 ± 11
Sex	Female	55 (44.4 %)
	Male	69 (55.6 %)
Recurrences		43 (34.7 %)
BMI [kg/m^2^]		30.0 ± 5.0
Hypertension		109 (87.9 %)
Diabetes		37 (29.8 %)
Coronary heart disease		49 (39.5 %)
Chronic heart failure		21 (16.9 %)
Chronic kidney disease		15 (12.1 %)
Peripheral artery disease		8 (6.5 %)
Hypercholesterinemia		56 (45.2 %)
Smoking		29 (23.4 %)
ECV		68 (55.6 %)
Ablation		55 (44.4 %)
Cryoablation		32 (25.8 %)
RFA		23 (18.5 %)
Antihypertensives [per patient]		2.6 ± 1.4
ACE inhibitors		39 (31.5 %)
AT1 antagonists		51 (41.1 %)
MRA		18 (14.5 %)
Calcium antagonists		38 (30.1 %)
Thiazides		7 (5.6 %)
Beta blockers		103 (83.1 %)
Alpha-2 agonists		2 (1.6 %)
Alpha-1 antagonists		14 (11.2 %)
Loop diuretics		49 (40.0 %)
Neprylsin inhibitors		3 (2.4 %)
Statins		85 (68.5 %)
Ezetimibe		27 (21.8 %)
Bempedoic acid		7 (5.6 %)
PCSK-9 inhibitors		1 (0.8 %)

**Fig. 1:**
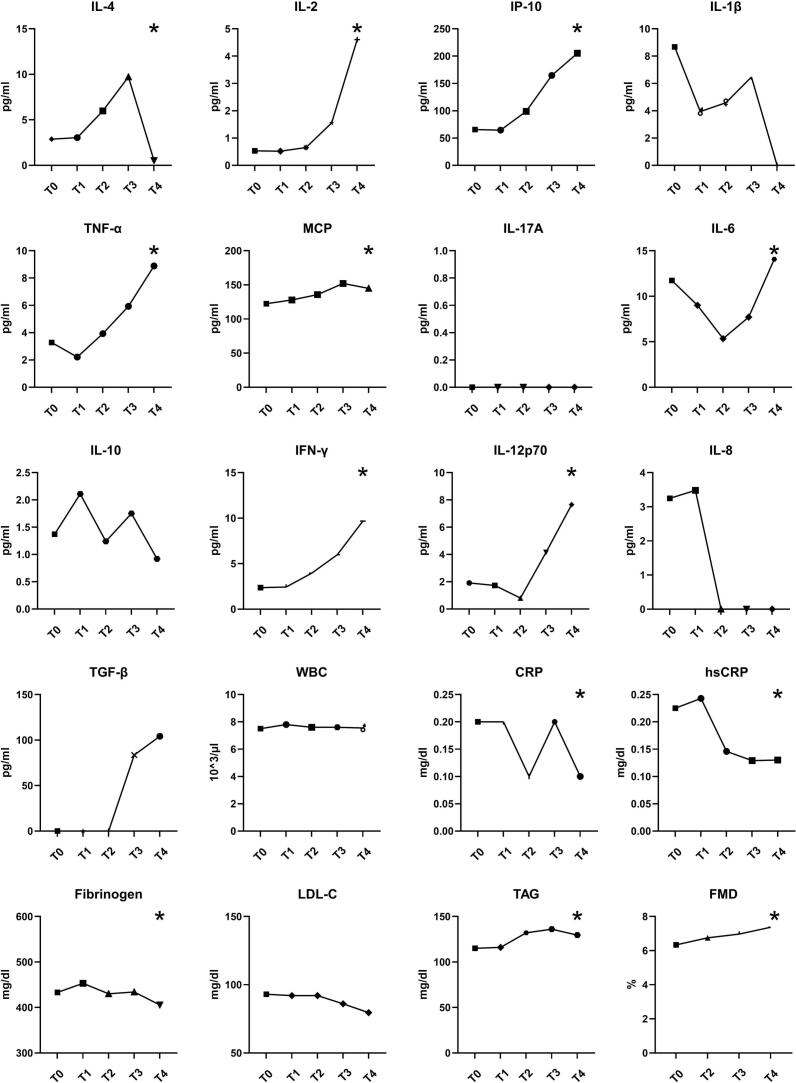
Cytokine and laboratory results for patients without recurrence of AF from T0 through T4 displayed as median, for reasons of visibility without range. Significant results in Friedman-Tests are marked with “*”. P < 0.05 was considered significant.

The analysis of variance (Friedman-test) revealed multiple significant differences in cytokine levels and laboratory parameters over the course of follow-up, as described in detail in in Fig. 1 (cohort without recurrence of AF). Specifically, significant changes were observed for IL-4 (p < 0.001), IL-2 (p < 0.001), IP-10 (p < 0.001), TNF-α (p < 0.001), MCP-1 (p < 0.001), IL-6 (p = 0.003), IFN-γ (p = 0.020), IL-12p70 (p = 0.004), CRP (p < 0.001), hsCRP (p < 0.001), fibrinogen (p < 0.001), triglycerides (TAG) (p < 0.001), and FMD (p = 0.017) from baseline (T0) through the 9-month follow-up (T4). When comparing baseline (T0) directly with the 9-month follow-up (T4), significant increases were found in IL-2 (0.5 (0–5.2) vs. 9.7 (2.3–32.4) pg/ml, p < 0.001), IP-10 (65.4 (35.5–117.3) vs. 205.4 (146.4–257.7) pg/ml, p < 0.001), TNF-α 3.3 (0–14.3) vs. 8.9 (0.8–38.4) pg/ml, p < 0.001), MCP-1 (124.8 (95.0–167.4) vs. 144.9 (87.4–196.3) pg/ml, p < 0.001) and IL12p70 (1.9 (0–15.9) vs. 7.7 (0–37.6) pg/ml, p < 0.001). Conversely, hsCRP (0.2 (0.1–0.5) vs. 0.1 (0.1–0.3) mg/dl, p = 0.002), IL-8 (3.3 (0–19.9 vs. 0 (0–63.6) mg/dl, p < 0.001) and fibrinogen levels (433.0 (390.5–520.0) vs. 405.5 (363.0–466.5) mg/dl, p = 0.003) decreased significantly. FMD measurement was assessed in all patients and improved significantly from T0 to T4 (6.3 (4.6–8.3) vs. 7.6 (5.1–8.8) %, p < 0.001). After successful rhythm control therapy FMD continued to improve numerically, yet not significantly from 6.8 (5.3–8.3) % at T2 to 7.4 (5.9–8.8) % at T4, (p = 0.27). Detailed longitudinal changes in inflammatory markers and vascular parameters across all follow-up time points, with all time points tested against each other, are illustrated in Supplementary Figs 1 and 2. In the linear mixed-effects model, FMD increased significantly from baseline to follow-up after adjustment for BMI, statin therapy, and antihypertensive medication (time effect: F = 15.77, p < 0.001). Adjusted estimated marginal means showed an increase in FMD from 6.35 % (95 % CI 5.81–6.88) at baseline to 7.42 % (95 % CI 6.93–7.92) at follow-up. Furthermore, differences (T4-T0) in FMD did not correlate with differences in cytokines.

**Fig. 2:**
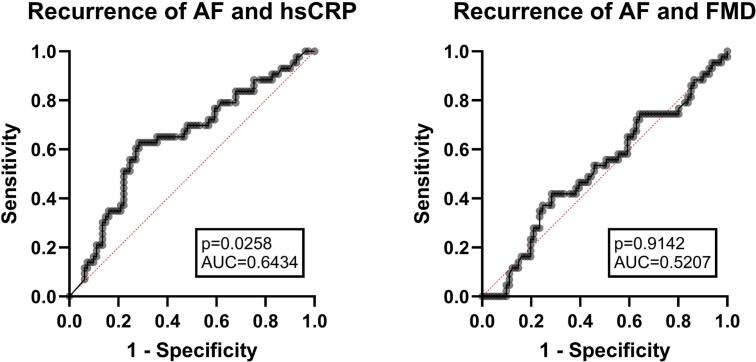
ROC Analysis of Recurrence of AF for high-sensitive c-reactive protein (hsCRP) and flow-mediated dilatation (FMD) at time of inclusion (T0). Level of significance is displayed as “p”. AUC = area under curve. P < 0.05 was considered significant.

Furthermore, we compared patients with and without recurrence of AF, as seen in Table 2. At baseline, patients who did not experience AF recurrence during follow-up showed significantly lower levels of IL-8 (0 (0–9.3) pg/ml vs. 3.3 (0.1–9.9) pg/ml, p = 0.004) and TGF-β (0 (0–0), 0 (0–151.9) p = 0.004) compared to those with recurrence. Conversely, hsCRP levels were significantly higher in patients with AF recurrence (0.5 (0.2–0.9) vs. 0.2 (0.1–0.5) mg/dl, p = 0.004). A correlation analysis (Pearson) showed a significant correlation between the recurrence of AF and hsCRP (r = 0.2, p = 0.023). Accordingly, a receiver operating characteristic (ROC) analysis showed weak predictability for AF recurrence (AUC = 0.61), indicating limited clinical utility of the tested biomarker for risk stratification, as displayed in Fig. 2. In a multivariate analysis including hsCRP, IL-8 and TGF-β, however, hsCRP was no longer independently associated with recurrence (OR 1.76; 95 % CI 0.89–3.58) and IL-8 was weakly and inversely associated with AF recurrence (OR 0.973; 95 % CI 0.937–0.998). We additionally analyzed cytokines and inflammatory markers in patients undergoing electrical cardioversion (ECV) and catheter ablation at baseline (T0) and at 9-month follow-up (T4), as shown in Supplementary Table 1. At baseline, the two groups already differed significantly with respect to IL-4, IL-1β, TNF-α, WBC, CRP, hsCRP, and FMD. At follow-up, IL-2 concentrations were significantly higher in patients treated with catheter ablation compared with those undergoing ECV (5.3 (2.7–10.6) vs. 2.4 (0.1–7.3) pg/ml, p = 0.016).

**Table 2:** Subgroup analysis for treatment group split into patients with and without recurrence of AF at time of inclusion (T0). Displayed as median with interquartile range 25–75 % and for ECV and ablation with relative distribution among the interventions. Level of significance (p). P < 0.05 was considered significant and are printed in bold.

	Recurrence of AF	No Recurrence of AF	p
IL-4 [pg/ml]	0 (0–38.9)	10.0 (0–36.5)	0.060
IL-2 [pg/ml]	0 (0–3.2)	0.5 (0–3.2)	0.172
IP-10 [pg/ml]	63.8 (35.5–97.6)	65.4 (35.5–117.3)	0.431
IL-1-β [pg/ml]	0 (0–35.6)	8.7 (0–33.0)	0.571
TNF-α [pg/ml]	2.1 (0–7.2)	3.3 (0–14.3)	0.023
MCP [pg/ml]	100.2 (73.8–165.7)	122.4 (93.4–165.3)	0.633
IL-17A [pg/ml]	0 (0–4.9)	0 (0–6.7)	0.063
IL-6 [pg/ml]	4.2 (0–15.5)	11.7 (0–30.8)	0.487
IL-10 [pg/ml]	0 (0–4.6)	1.4 (0–7.0)	0.600
IFN-γ [pg/ml]	0 (0–3.6)	2.4 (0–21.8)	0.445
IL-12p70 [pg/ml]	0 (0–11.3)	1.9 (0–15.9)	0.105
IL-8 [pg/ml]	0 (0–9.3)	3.3 (0–19.9)	**0.004**
TGF-β [pg/ml]	0 (0–0)	0 (0–151.9)	**0.004**
WBC [10^3^/µl]	8.0 (6.1–9.2)	7.5 (6.4–9.3)	0.770
CRP [mg/dl]	0.4 (0.2–0.8)	0.2 (0.1–0.4)	0.244
hsCRP [mg/dl]	0.5 (0.2–0.9)	0.2 (0.1–0.5)	**0.030**
Fibrinogen [mg/dl]	461.0 (407.0–523.0)	433.0 (390.5–520.0)	0.210
LDL-C. [mg/dl]	85.0 (65.0–111.0)	93.0 (68.5–130.0)	0.120
TAG [mg/dl]	105.0 (84.0–154.0)	115.0 (85.5–168.0)	0.377
FMD [%]	6.1 (5.0–7.4)	6.3 (4.6–8.3)	0.331
ECV [total]	15 (22.1 %)	53 (77.9 %)	**0.048**
Ablation [total]	5 (8.9 %)	51 (91.1 %)

## 4 Discussion

This study investigated the impact of rhythm control therapy in patients with AF on systemic inflammation and endothelial function. A significant improvement in FMD was observed following rhythm therapy, indicating enhanced endothelial function. Noteworthy, patients with AF indeed exhibited impaired endothelial function prior to the intervention. In addition, several cytokine concentrations changed significantly over time. However, these changes did not follow a consistent pathophysiological pattern, as both pro- and anti-inflammatory cytokines showed increases as well as decreases during follow-up.

In our study hsCRP was associated with only a mild positive correlation and weak predictive value for AF recurrence, as indicated by our ROC analysis. Cytokine analyses following rhythm control therapy revealed heterogeneous results. While a reduction in pro-inflammatory cytokine concentrations was anticipated based on existing literature, levels of IL-2, IP-10, IL-12p70, MCP-1 and TNF-α increased significantly from baseline (T0) to the final follow-up (T4). Our findings are consistent with the heterogeneous results reported in prior trials on AF recurrence. While Wazni et al., Malouf et al., and Watanabe et al. demonstrated higher rates of AF recurrence in patients with elevated CRP prior to rhythm-control therapy, Korantzopoulos et al., Cosgrave et al., and Buob et al. did not observe such an association [[22], [23], [24], [25], [26], [27]]. More recently, Bastiancic et al. even reported a protective effect of TNF-α on AF recurrence, further underscoring the heterogeneity of findings and suggesting that elevated pro-inflammatory cytokines, in some contexts, may exhibit a beneficial effect [28]. Our immunologically diverse results may be explained by the varying half-lives and release kinetics of individual cytokines, resulting in different temporal concentration patterns. Additionally, restoration of sinus rhythm may shift the cytokine milieu—reducing certain stimuli such as shear stress and atrial stretch, while simultaneously introducing others, such as myocardial remodeling or procedural tissue injury. Divergent cytokine responses may also reflect patient-specific factors and individual comorbidities, which can influence both baseline cytokine expression and the magnitude of inflammatory response following rhythm control.

A key finding of this study is the heterogeneous behavior of systemic inflammatory markers following successful rhythm control. While hsCRP, IL-8, and fibrinogen declined over time, several cytokines commonly considered pro-inflammatory, including IL-2, IP-10, IL-12p70, MCP-1, and TNF-α, showed significant increases. These divergent observations indicate that restoration of sinus rhythm does not result in a uniform suppression of systemic inflammation and underscore the complexity of immune regulation in persistent atrial fibrillation.

This heterogeneity may be explained by differences in cytokine origin, half-life, and regulatory pathways, leading to asynchronous temporal patterns despite rhythm stabilization. In addition, rhythm control may reduce atrial stretch and shear stress while simultaneously promoting longer-term immune remodeling related to myocardial repair and neurohumoral adaptation. Consequently, increases in certain cytokines may reflect compensatory or reparative processes rather than persistent pathological inflammation. Patient-specific factors, including comorbidities and background pharmacotherapy, are also likely to modulate inflammatory profiles and may outweigh rhythm-related effects.

Although procedural inflammation after catheter ablation could theoretically contribute to cytokine elevation, this appears unlikely in our cohort, as inflammatory marker trajectories – besides an increase in IL-2 – did not differ between ablation and electrical cardioversion and remained heterogeneous throughout the nine-month follow-up. Together, these findings suggest that the observed cytokine patterns primarily reflect complex, patient-specific immune dynamics rather than a uniform inflammatory response to rhythm control therapy.

As previously reported, several studies have assessed endothelial function using FMD. In a large *meta*-analysis including over 5000 patients Yoichi et al. described an association between impaired FMD and future cardiovascular events, suggesting FMD as an early marker of vascular health [29,30]. Consistent with our findings, Kanazawa et al. and Yoshino et al. demonstrated that patients with AF exhibit impaired endothelial function compared to those in sinus rhythm. Notably, both studies observed significant improvement in endothelial function following restoration of sinus rhythm, achieved via catheter ablation [31,32]. From a pathophysiological perspective, the restoration of sinus rhythm and the subsequent elimination of AF may improve endothelial function through several mechanisms:

1. Normalization of Shear Stress: AF induces irregular flow patterns due to inconsistent cardiac ejection, resulting in oscillatory shear stress, increased oxidative stress, and reduced nitric oxide (NO) bioavailability. Restoration of sinus rhythm re-establishes laminar flow, thereby enhancing endothelial NO responsiveness and reducing oxidative burden [33].
2. Improved cardiac output: AF impairs atrial contribution to ventricular filling, leading to reduced stroke volume and cardiac output. Sinus rhythm restores coordinated atrial contraction, improving hemodynamics and tissue perfusion [34].
3. Inflammation: AF is associated with elevated levels of pro-inflammatory cytokines such as CRP and IL-6, potentially driven by atrial stretch, endothelial activation, and impaired circulation. There is robust evidence, that inflammation is associated with an impairment of endothelial function. In this context, a highly published milestone study showed that treatment of periodontitis led to a reduction of systemic inflammation with consecutive improvement of FMD [35]. Thus, a reduction of inflammation constitutes a promising candidate mechanism to explain the improvement in FMD following rhythm control in AF.

In the present study, however, no consistent reduction in systemic inflammatory markers was observed. One possible explanation is that local tissue injury associated with ablation may transiently increase inflammatory biomarkers. Nevertheless, the nine-month follow-up period argues against this interpretation. Moreover, patients treated with ECV would be expected to demonstrate reductions in cytokine levels, which were not detected. In our subgroup analysis, trajectories of inflammatory biomarkers did not differ between patients undergoing ablation and those treated with ECV. Collectively, these findings do not support the hypothesis that improvement in vascular function following rhythm control in AF is mediated by reductions in inflammation. Instead, the observed improvement in endothelial function is more likely to reflect enhanced hemodynamic stability and improved nitric oxide–dependent vascular responsiveness, which may represent one mechanistic link to the broader cardiovascular benefits observed in patients with durable rhythm control. In line with this interpretation, longitudinal changes in FMD did not correlate with changes in any of the measured cytokines. These findings highlight the complex interplay between rhythm control, vascular function, and the inflammasome in AF.

The strengths of this study lie in the comprehensive cytokine analyses performed in patients with persistent atrial fibrillation, which for the first time were combined with an assessment of endothelial function by FMD. We investigated a real-world cohort of patients requiring rhythm control therapy and included both those undergoing ECV and those treated with catheter ablation, with an extensive follow-up period of nine months.

This study has several limitations. First, the absence of a control group of patients with persistent atrial fibrillation managed conservatively by rate control alone limits causal attribution of the observed improvement in endothelial function specifically to rhythm restoration. Nonetheless, the consistent and sustained improvement in FMD over nine months, together with the lack of a uniform anti-inflammatory response, suggests that restoration of regular cardiac rhythm and hemodynamic normalization are likely key contributors. Although our cohort of 124 patients represents one of the largest to date investigating atrial fibrillation, vascular function, and cytokine responses to rhythm control therapy, the prospective, non-randomized observational design is susceptible to confounding factors, particularly with regard to treatment allocation (ECV vs. catheter ablation). Cytokine measurements were collected at predetermined follow-up intervals, which may have missed transient cytokine peaks due to variable half-lives and release kinetics. Moreover, the inherently invasive nature of catheter ablation induces a localized inflammatory response, which may contribute to the heterogeneous cytokine patterns observed following rhythm control therapy. While all FMD assessments were performed by a single experienced operator to ensure consistency, the potential for operator bias cannot be entirely excluded.

In conclusion, the study confirms that rhythm control in persistent AF is associated with a significant improvement in FMD and modifies systemic inflammatory profiles. However, the inflammatory response is heterogeneous and appears to be influenced by individual patient factors. These findings do not support the concept of a bidirectional relationship between AF and systemic inflammation. Furthermore, despite the well-documented association between endothelial dysfunction and inflammation, the observed improvement in FMD following rhythm control is likely mediated predominantly by hemodynamic restoration rather than by anti-inflammatory effects.

## CRediT authorship contribution statement

**Maximilian Seidel:** Writing – original draft, Visualization, Methodology, Formal analysis. **David Bogdahn:** Data curation. **Felix S. Seibert:** Formal analysis. **Moritz Anft:** Validation, Project administration. **Sarah Skrzypczyk:** Validation, Data curation. **Ulrik Stervbo:** Visualization, Validation, Investigation. **Eva Kohut:** Investigation, Data curation. **Kamil Rosiewicz:** Visualization, Software, Formal analysis. **Benjamin Sasko:** Writing – review & editing, Project administration. **Christian Ukena:** Writing – review & editing, Project administration. **Nina Babel:** Writing – review & editing, Resources, Methodology, Investigation, Conceptualization. **Timm H. Westhoff:** Writing – review & editing, Validation, Supervision, Resources, Methodology, Conceptualization.

## Declaration of competing interest

The authors declare that they have no known competing financial interests or personal relationships that could have appeared to influence the work reported in this paper.

## Data Availability

Data will be made available on request.
