# Supplementary material for: Rhythm control in persistent atrial fibrillation improves endothelial function without uniform anti-inflammatory effects: A 9-month prospective cohort study
Source: Int J Cardiol Heart Vasc. 2026 Jan 23;63:101879. doi: 10.1016/j.ijcha.2026.101879 (PMC12860693; doi:10.1016/j.ijcha.2026.101879)
Supplement: Supplementary Data 1 [file mmc1.docx]

|  | T0 | | P | T4 | | P |
| --- | --- | --- | --- | --- | --- | --- |
|  | ECV (n=35) | Ablation (n=46) |  | ECV (n=35) | Ablation (n=46) |  |
| IL-4 [pg/ml] | 4.8 (0-22.2) | 15.3 (0.8-60.2) | **0.034** | 4.1 (0.3-26.7) | 13.0 (3.9-35.5) | **0.041** |
| IL-2 [pg/ml] | 0 (0-3.4) | 0.9 (0-5.5) | 0.259 | 2.4 (0.1-7.3) | 5.3 (2.7-10.6) | **0.016** |
| IP-10 [pg/ml] | 73.8 (31.6-147.1) | 55.5 (37.4-104.2) | 0.522 | 207.3 (149.7-269.1) | 201.2 (140.9-252.5) | 0.642 |
| IL-1-β [pg/ml] | 0 (0-21.8) | 15.6 (0-56.0) | **0.018** | 0.9 (0-22.4) | 0 (0-21.6) | 0.963 |
| TNF-α [pg/ml] | 0.3 (0-12.5) | 6.5 (0-15.4) | **0.090** | 1.8 (0-23.1) | 15.2 (3.6-40.8) | **0.028** |
| MCP [pg/ml] | 122.4 (93.5-175.9) | 124.9 (99.4-165.0) | 0.802 | 128.7 (80.9-204.6) | 151.1 (100.3-196.1) | 0.524 |
| IL-17A [pg/ml] | 0 (0-4.6) | 0 (0-10.8) | 0.556 | 0 (0-13.3) | 0 (0-20.0) | 0.592 |
| IL-6 [pg/ml] | 11.2 (0-31.5) | 13.6 (0-36.9) | 0.620 | 5.7 (0.6-34.6) | 17.3 (5.1-43.5) | 0.095 |
| IL-10 [pg/ml] | 0.2 (0-5.8) | 1.8 (0-8.6) | 0.350 | 0.5 (0-2.6) | 0.9 (0-9.2) | 0.406 |
| IFN-γ [pg/ml] | 0.3 (0-8.6) | 4.6 (0-35.5) | 0.083 | 5.2 (0-14.2) | 10.2 (0-32.2) | 0.084 |
| IL-12p70 [pg/ml] | 1.3 (0-14.0) | 6.0 (0-20.1) | 0.273 | 2.8 (0-15.1) | 9.8 (0-40.3) | 0.107 |
| IL-8 [pg/ml] | 1.7 (0-16.2) | 5.0 (0-25.2) | 0.243 | 0 (0-48.2) | 21.8 (0-73.6) | 0.154 |
| TGF-β [pg/ml] | 0 (0-88.9) | 27.2 (0-159.8) | 0.155 | 170.2 (0-230.0) | 0 (0-218.3) | 0.382 |
| WBC [10^3^/µl] | 8.0 (6.8-11.1) | 7.0 (6.0-8.6) | **0.023** | 8.2 (6.8-8.8) | 7.1 (5.6-8.7) | **0.037** |
| CRP [mg/dl] | 0.2 (0.1-0.8) | 0.1 (0.1-0.3) | **0.025** | 0.2 (0.1-0.3) | 0.1 (0.1-0.2) | **0.046** |
| hsCRP [mg/dl] | 0.2 (0.1-0.8) | 0.2 (0.1-0.3) | **0.026** | 0.2 (0.1-0.3) | 0.1 (0.1-0.2) | 0.095 |
| FMD [%] | 5.7 (4.2-6.9) | 6.9 (4.9-8.5) | **0.014** | 6.6 (5.0-8.7) | 8.2 (6.4-9.2) | 0.094 |

**Table 1** Cytokine concentrations, inflammatory markers and FMD at T0 and T4 for electrical cardioversion (ECV) vs. ablation. Displayed as median with interquartile range (25-75%). P<0.05 was regarded significant.


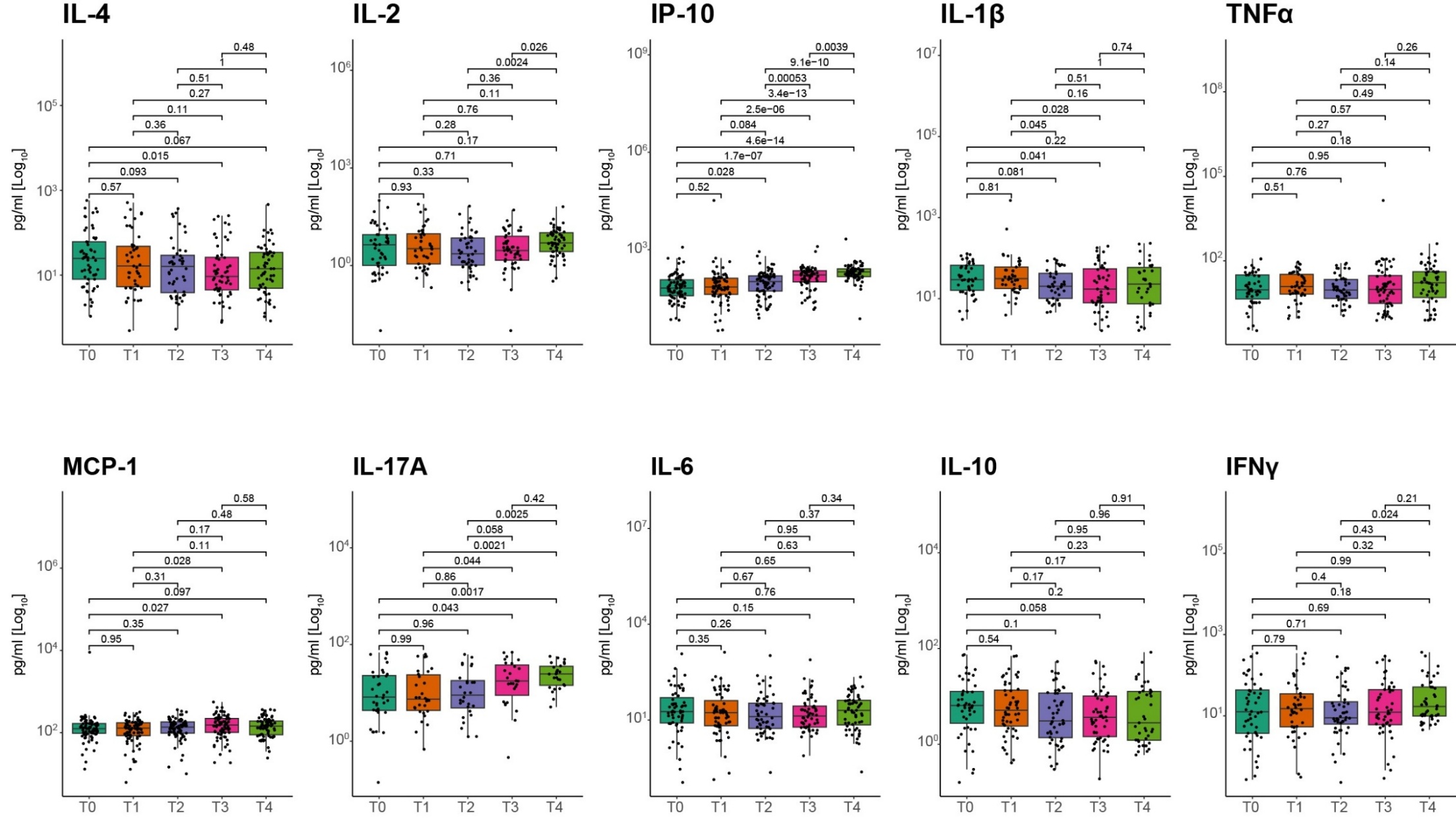


**Supplementary Figure 1**: Part 1 of 2: Cytokine analyses displayed as boxplots including median with interquartile range (25^th^ – 75^th^ percentile) and minimum and maximum value in the data. Level of significance of Wilcoxon-Test is displayed for the corresponding time points T0 through T4.


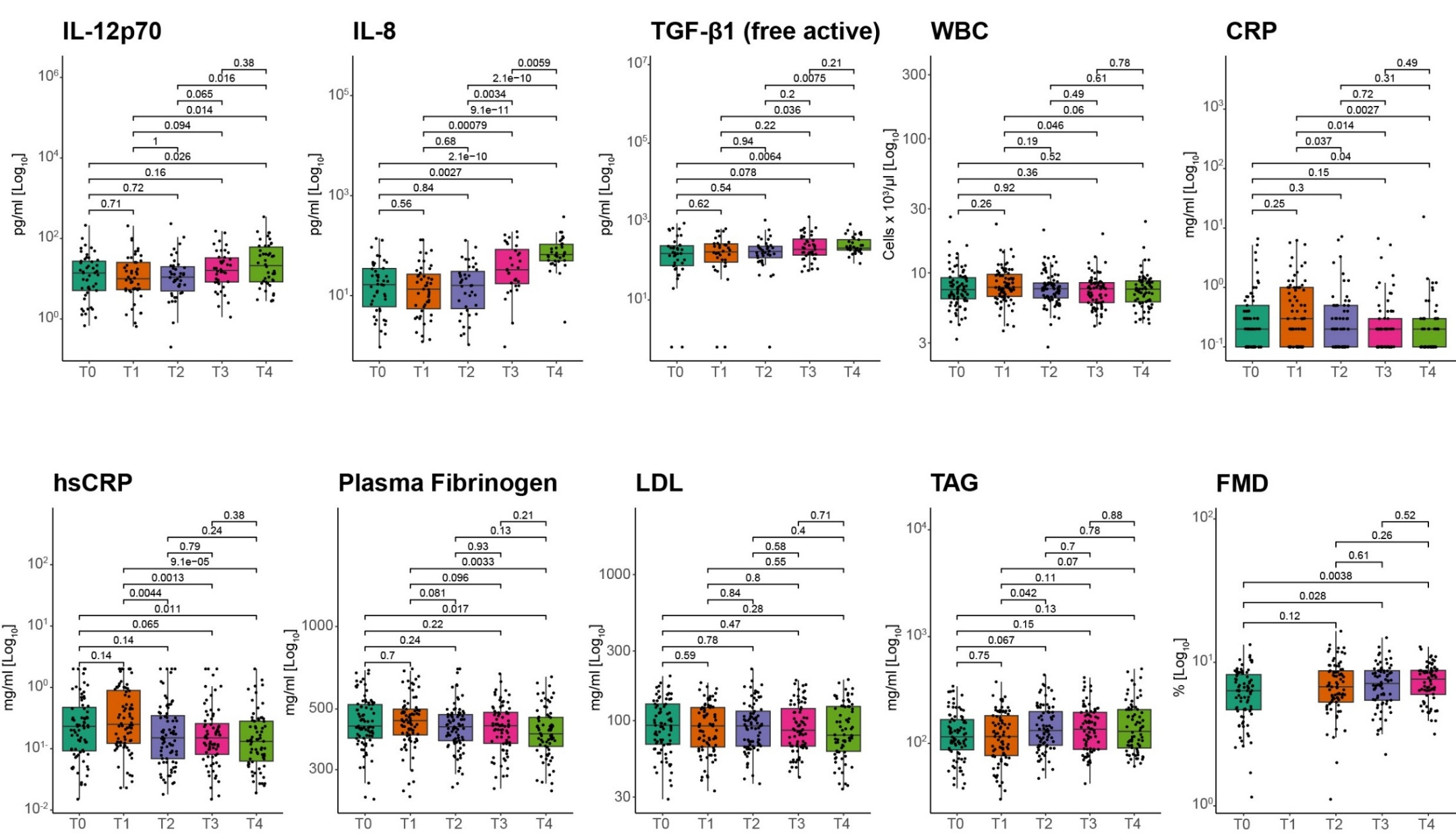


**Supplementary Figure 2**: Part 2 of 2: Cytokine analyses displayed as boxplots including median with interquartile range (25^th^ – 75^th^ percentile) and minimum and maximum value in the data. Level of significance of Wilcoxon-Test is displayed for the corresponding time points T0 through T4.
